# Scaling up services for mental and neurological disorders in low-resource settings

**DOI:** 10.1016/j.inhe.2009.02.002

**Published:** 2009-09

**Authors:** Vikram Patel, Digvijay Singh Goel, Rajnanda Desai

**Affiliations:** a London School of Hygiene and Tropical Medicine, Keppel Street, London WC1E 7HT, UK; b Southland Hospital, Invercargill 9812, New Zealand; c Government of Goa, India

**Keywords:** Mental disorders, Neurological disorders, Health services, Scaling up, Developing countries, India

## Abstract

Mental and neurological disorders (MNDs) account for a large, and growing, burden of disease in low- and middle-income countries. Most people do not have access to even basic health care for these disorders. Recent evidence shows that task-shifting to non-specialist community health workers is a feasible and effective strategy for delivery of efficacious treatments for specific MND in low-resource settings. New global initiatives, such as the WHO's mental health Gap Action Program, are utilizing this evidence to devise packages of care for specific MNDs. This paper describes a plan that seeks to integrate the evidence on the treatment of specific MNDs, based on a task-shifting paradigm, for scaling up services for MNDs at the level of a defined population. The plan was developed by a state government in India in collaboration with technical partners, as a model District Mental Health Program for India's National Mental Health Program.

## 1 Background

The *Global Burden of Disease and Risk Factors*^1^ is a widely used benchmark to assess, and compare, the burden posed by various health conditions in each region of the world. According to this report, mental and neurological disorders (MNDs) account for 9.8% of the total burden of disease in low- and middle-income countries (LAMICs). Self-inflicted injuries account for 1.5% of all deaths in LAMICs, and this is likely to be an underestimate due to reporting biases. There are also considerable regional variation in burden of MNDs; for example, self-inflicted injuries account for over 2% of total deaths in Eastern Europe and Central Asia, making them the fifth leading cause of mortality in these LAMICs. Unipolar depressive disorder is the leading cause of ‘years lived with a disability’; two other mental disorders appear in the leading 10 causes: schizophrenia and alcohol-use disorders. It is in this context, no less than one of the biggest global health crises of our times, that a group of academics produced the landmark *Lancet* series on global mental health. The series reviewed the evidence, focusing on mental disorders in LAMICs. The major messages arising from the series are listed in Table 1.

**Table 1:** The messages of the *Lancet* series on global mental health.

• Mental disorders are so inextricably linked with other public health priorities (such as HIV/AIDS, maternal and child health and diabetes) that there is ‘no health without mental health’.^27^
• Resources for mental health care are extremely scarce in most low- and middle-income countries (LAMICs); the resources that do exist are very inequitably distributed and inefficiently utilized.^2^
• There is growing evidence on the efficacy and cost-effectiveness of pharmacological and psychosocial treatments for many mental disorders.^18^
• There are several barriers to scaling up mental health services, notably the stigma associated with mental disorders, poor human resource capacity, weak public health perspective among the mental health professions, weak general health-care systems, lack of political will, poor governance of the health system and lack of a clear and consistent advocacy message.^28^

In spite of this evidence, MNDs in LAMICs are, arguably, the most neglected global health problem on virtually any metric we choose. The ratio of burden to resources available for mental health care in LAMICs is extremely inequitable, perhaps one of the worst among all major health domains.^2^ Unsurprisingly, the ‘treatment gaps’ for people with MNDs exceed 50% in most countries and approach 90% in some.^3^, ^4^ Large numbers of people with MND suffer some of the most appalling human rights abuses witnessed in modern times, often perpetrated within the institutions run to care for them.^5^ Family members, left often without any access to care, are forced to rely on restraints and other degrading practices to manage disturbed behaviours.^6^ The vast majority of those who suffer from MNDs with no obvious externally apparent symptoms (for example, those with depression or alcohol-use disorders) are simply ignored altogether.

The *Lancet* series ended with a call to action to scale up services for people with mental disorders.^7^ A number of initiatives have been launched in response to this call to action.^8^ A crucial goal is to describe packages of care for specific mental disorders. The WHO's new Mental Health Gap Action Program (mhGAP) specifically aims to develop packages of care for seven mental, neurological and substance-abuse disorders and for suicide.^9^ The World Psychiatric Association has launched an initiative to assess psychiatrists’ perspectives on delivery of evidence-based treatments for mental disorders across the continuum of life, with a focus on mechanisms that aim to achieve equity of coverage in low-resource settings. The journal *PLoS Medicine* will soon publish a series of articles on packages of care for six MNDs. The Movement for Global Mental Health^10^ has launched a website from which unpublished packages of care can be disseminated.^11^

All these initiatives focus on either specific disorders or specific demographic groups. The key challenge remains how these various packages can be integrated across disorder categories and demographic groups and, furthermore, how these can be integrated within existing health-care programmes. In this article, the evidence available so far is utilized to propose such an integrated model. The article will first consider how disorders can be grouped based on shared clinical, epidemiological and health-service considerations. Next, the delivery of the packages of care for each group of disorders will be considered based on a primary-care delivery system serving a defined population or ‘health district’.^12^ This article is based on the development of a District Mental Health Program (DMHP) proposal developed by the authors, on behalf of the government of a state in India, in response to the National Mental Health Program of India.

## 2 Integrating clinical syndromes

Modern classifications of MNDs, such as ICD10,^13^ have more than a hundred diagnostic categories. Even shorter, pragmatic classifications, such as the primary-care version of ICD10, contain more than 20 mental disorder diagnostic categories. Many of these categories can be merged, for health-service planning, based on similar epidemiological and clinical characteristics.^14^ In this article, we have proposed a broad classification of MNDs for reasons shown in Table 2.

**Table 2:** A pragmatic classification of mental and neurological disorders (MNDs).

	Common MNDs	Severe MNDs
Clinical syndromes	• Depression/anxiety (or CMDs)	• Psychotic disorders
	• AUDs	• Dementias
		• Epilepsy
		• Mental retardation
		• Strokes
Presenting clinical features	• Present mainly in general or primary health care	• Low use of primary care
	• Somatic complaints dominate	• Help-seeking often precipitated by acute events, e.g. disturbed behaviour, loss of neurological function
	• Most people do not consider their illness an MND	• Help-seeking often through indigenous providers or directly through specialist services if available
Epidemiological characteristics	• Common (@5% of population)	• Less frequent
	• Risk is heavily influenced by social determinants	• Genetic and biological environmental determinants
	• Often co-morbid with each other	• Often co-morbid with each other
Detection	• Brief screening questionnaires	• First stage through key informants or emergency assessment in crisis situations; confirmation by trained health worker
Course and outcome	• Many will recover, but relapses common	• Chronic course
		• Poor outcomes for dementia, strokes
Opportunities for integration with other health programmes	• Chronic diseases	• Disability programmes
	• Maternal and child health	• Chronic diseases
	• HIV/AIDS	• School health
	• School health	

AUD: alcohol-use disorder; CMD: common mental disorder.

Based on this pragmatic classification, a substantial body of epidemiological research shows that approximately 5% of the adult and child populations suffer from an MND that needs clinical intervention. The commonest MNDs in adults are common mental disorders (CMDs) and alcohol-use disorders (AUDs); CMDs are the commonest MNDs affecting women, whereas AUDs are the commonest MNDs in men. The most frequent MNDs affecting children are mental retardation, epilepsy and mood disorders. About 1 in 5 adults attending primary care suffer from a CMD. There are some contextual variations in these epidemiological findings – for example in the exact prevalence rates – but these estimates are of general use for health-district planning. Most MNDs are more frequent among the less educated and poor. Social factors, in particular economic deprivation and violence, are strongly associated with CMD. Most people with an MND do not receive evidence-based treatments; the treatment gap for CMD and epilepsy, for example, is over 80% in some contexts.^15^ Severe MNDs are associated with considerable stigma and discrimination in levels of society, leading to exclusion from participation in education and employment, and pose a huge burden on caregivers.

This pragmatic classification of MNDs is the basis for designing the intervention framework of the DMHP. Thus, the management of common MNDs relies principally on early detection through routine or high-risk group screening of general or primary health-care attenders. The management of severe MND relies principally on active case-finding and follow-up in the community and adequate provision for in-patient care for severe presentations. Details of the delivery of evidence-based interventions are presented below. The DMHP presented in this article is primarily intended to inform the development of an integrated service-delivery plan for a specific district in India; however, this programme can also serve as a paradigm that can be adapted for use elsewhere.

## 3 Values and objectives of the District Mental Health Program

The values of the proposed DMHP plan are consonant with the themes of India's *National Mental Health Policy: Vision 2020* document,^16^ which envisaged:

• *accessibility* of at least basic MND care facilities within the community to as large a section of the population as possible;
• *affordability* of MND services with regard to the initial capital cost as well as recurring expenditure (including that on essential drugs) to accord with limited resources and low income levels of the consumer population;
• *adaptability* to the widely varying geographical, socio-cultural and economic mosaic characteristic of India's health system;
• *acceptability* of MND health care by the target population in the context of low levels of literacy, ignorance, superstition, economic backwardness and lack of empowerment of women, adolescents and children;
• *assessment* of performance at the ground level through continuous monitoring, audit and periodic review and introduction of necessary corrective measure and relevant feedback for future planning.

The objectives are consonant with the draft 11th National Mental Health Program^17^ and the Movement for Global Mental Health:^10^ to scale up services for MND based on the best available evidence of effectiveness and protection of the human rights of persons affected. Ultimately, the plan aims to:

• promote early recovery in people suffering from MNDs;
• promote the health and quality of life of people suffering from MNDs;
• promote inclusion and reduce discrimination experienced by; to people with MNDs;
• reduce premature mortality associated with MNDs.

## 4 The District Mental Health Plan

The starting point for the District Mental Health Plan is the broad categorization of MNDs, as outlined earlier. The next step is the identification of effective treatments, on the basis of two key recent analyses: the *Disease Control Priorities Project*^15^ and the systematic review of treatments for mental disorders in the *Lancet* series.^18^ These treatments are summarized in Table 3. The focus is on individual level interventions within the domain of the health sector (therefore, structural or policy interventions, such as taxation on alcohol use are not included in this plan).

**Table 3:** Personal/individual interventions for mental and neurological disorders (MNDs).

Disorder	Primary treatment	Other interventions
Mental retardation	• Psychosocial stimulation to promote early child development	• Preventive interventions, for e.g. iodine supplementation, prenatal and newborn screening, improved maternal care, etc.
	• Community-based rehabilitation	• Caregiver support
Common mental disorders	• Antidepressant medication	• ECT for severe depression
	• Brief psychological treatment	
Alcohol-use disorders	• Brief psychological treatment	• Alcohol withdrawal management
		• Anti-craving medications (e.g. acamprosate)
		• Caregiver support
		• Support groups (e.g. AA)
Schizophrenia	• Antipsychotic medication	• Residential or daycare
	• Community-based rehabilitation	• Caregiver support
Bipolar disorder	• Mood stabilizer medication	• Caregiver support
	• Antipsychotic medication	
Epilepsy	• Anticonvulsant medication	• Acute management for status epilepticus
		• Community-based rehabilitation
Dementia	• Caregiver support	• Residential or daycare
	• Antipsychotic medication	
Stroke	• Improved management of risk factors (e.g. hypertension)	• Acute stroke management
	• Community-based rehabilitation	• Platelet antiaggregants

AA: Alcoholics Anonymous; ECT: electroconvulsive therapy.

There is much weaker evidence on scaling up these efficacious treatments in LAMICs, but there is a growing evidence base on the effectiveness of task-shifting for the care of a variety of MNDs in such settings.^15^, ^18^ Based on these pieces of evidence, the plan concentrates on the delivery of services by four types of human resources (Table 4): the health district manager, the programme leader, the health counsellor and community volunteers/service users.

**Table 4:** Key human resources and roles for the District Mental Health Plan.

Key human resource	Basic qualification	Roles
Health district manager/Primary Health Centre Health Officer	Public health	• Overall leadership of the district health programme
		• Ensuring adequate resources and integration of mental health programme within all health activities
		• Ensuring no discrimination against people with MNDs within the health-care sector
Mental Health Program Leader	Psychiatrist or other mental health specialist with training and licensing for medication use; or general physician or other non-specialist practitioner with training in mental health diagnostics and medication use	• Overall leadership of mental health programme
		• Diagnosis of severe MND
		• Initiation of pharmacological treatments for MND
		• Reviews of pharmacological treatment
		• Training and supervision of health counsellors
		• Setting benchmarks and monitoring progress
		• Refining and revising programme activities based on evaluation
		• Ensuring that people with MND receive equitable care for physical health problems
		• Advocacy and participation in community activities to protect human rights and build mental health literacy
Health counsellors	College graduates appropriately trained to fulfil roles	• Screening for common MNDs
		• Community surveillance for detection and referral of probable severe MNDs
		• Psychological interventions for individuals and families
		• Enabling self-help groups
		• Networking with other helping agencies (e.g. special schools)
Service users/volunteers	Non-formal members of the mental health team	• Community surveillance for detection and referral of probable severe MND
		• Provision of social support to affected individual and families
		• Enabling self-help groups

MND: mental and neurological disorder.

The primary additional human resources to be employed through ring-fenced mental health programme funds are the Mental Health Program Leader (MHPL) and the health counsellors. We estimated the requirement for one MHPL to cover a population of about 500 000–750 000 people, i.e. the population of the selected health district. Within this district, there are a number of primary health centres (PHCs). The MHPL takes overall responsibility for all clinical aspects of the programme. He/she would visit each PHC on a regular basis and would be responsible for monitoring the quality of care, building capacity, supervising PHC staff and assessing complex cases. He/she would also be responsible for training and supervising health counsellors. Finally, they would be primarily responsible for ensuring that people with MNDs are not discriminated against in the health sector and that their physical health needs are equitably addressed.

Health counsellors are the ‘frontline’ MND care providers based in each PHC. They are full-time health workers, modelled on the health counsellor model of the Manas Program for depression;^19^ the Ashagram Community Mental Health Programme for people with schizophrenia;^20^ and the Home Care Assistant Programme for caregivers of persons with dementia.^21^ They are local residents who have passed college and have been trained to detect and provide a range of psychosocial treatments for MND. These will include screening PHC attenders to detect depression and alcohol abuse, brief psychological treatments and psychoeducation, regular yoga courses and adherence management for those taking medication. They are also trained to detect probable cases of severe MND in community settings and refer them to the PHC for diagnostic assessment, support families and caregivers of people with severe MND, and promote inclusion and challenge discrimination against people with MND. Each PHC will have an allocation of four health counsellors, one of whom will be appointed to take overall charge of the implementation of the programme in that PHC. They will be trained by the MHPL in a four month structured training programme based on the curricula developed for the projects cited above. Regular supervision and refresher courses will be held, which will be combined with mental health promotion exercises for these staff.

The levels of care delivery are those based on the usual primary-care model, and adopted by India's National Rural Health Mission (Table 5).

**Table 5:** Delivery of the integrated MND plan through the primary care system.

Level of care	Key care provider	Goals	Interventions
Community/households	• Health Counsellor	• Detection and home-based care and rehabilitation of severe MND	• Mental health assessments
	• Service Users/families	• Increasing awareness	• Psychosocial stimulation (for infants)
	• Volunteers	• Strengthening social support and inclusion	• Detection and referral
		• Promoting adherence and recovery	• Psycho-education
			• Facilitating social welfare benefits
			• Adherence management
			• Review of progress
			• Self-help groups
Primary Health Centre and district general hospital	• Health counsellors	• Detection of common MNDs	• Mental health assessments
	• Mental health		
	• Programme leaders		
		• Confirmation of diagnosis of severe MND	• Provision of appropriate medications
		• Monitoring of individual case management plans for all MNDs	• Provision of psychological treatments and yoga
		• Inpatient management of acute or severe MNDs	• Clinical assessments and case note reviews
			• Brief hospital admissions, for inpatient management of emergencies: suicide attempt, acute psychosis, alcohol withdrawal, status epilepticus, etc.
Other community agencies, e.g. NGOs	• NGO workers	• Mental health promotion and prevention of MND	• Mental health promotion
	• Service Users/families	• Promoting recovery and reintegration of people with MND	• Community-based rehabilitation
	• Volunteers		• Self-help groups
			• Advocacy
			• Daycare and residential care

MND: mental and neurological disorder; NGO: non-governmental organization.

Apart from these services aimed at detection and treatment of MNDs, the plan will also implement two related activities:

• IEC programmes for raising awareness about MND and discrimination based on successful interventions for improving mental health literacy and challenging stigma;^22^, ^23^
• school mental health programmes based on the WHO Life Skills package, adapted for use in India, aimed to train school teachers and peers.^24^ The PHC based health counsellors will be assigned the responsibility to carry out this programme in schools in the health district.

## 5 Project management and monitoring

The programme would be monitored by an inter-sectoral advisory board comprising representatives of the government health agency, non-governmental organizations and consumers. This group would meet once every quarter and review the progress of the DMHP, monitor the attainment of targets and, based on these findings, advise on strategy directions for the next reporting period. The group would also report to relevant national health information systems, such as the Noncommunicable Disease Control Program. The group will agree, with the DMHP implementing team, on a set of clear and quantifiable benchmarks (including a time-frame for achieving the pre-determined targets) for facilitating monitoring and audit of the various programme components. Some examples of key indicators that would be used to monitor the DMHP, derived from recent WHO and other reports,^7^, ^25^ are listed in Table 6. These include a combination of input indicators (such as the number of new human resources trained), process indicators (such as numbers of patients diagnosed/treated) and outcome indicators (such as relapse rates).

**Table 6:** Indicators for monitoring progress in the District Mental Health Program.

• The number of new human resources trained and located in the PHCs.
• The number of cases of long-standing (>1 year duration) who had previously not been in contact with health services now in contact.
• The estimated coverage of core MNDs based on expected number of cases and the number identified and in care.
• The number and type of prescriptions for psychotropic medicines delivered through PHCs.
• The number/proportion of people living below the poverty line, or from socially or economically disadvantaged sections of the community, who access the DMHP.
• The number/proportion of people from different economic strata, and gender, who are diagnosed with an MND, who receive mental health interventions or referrals.
• The number of people with MND receiving community-based care/rehabilitation services.
• The evaluation by users of health services of the quality of care (for example, adequacy of time spent with the health worker, satisfaction with the explanation given regarding the symptoms).
• Relapses in people who have had contact with the programme.
• The number of suicides each year.

DMHP: District Mental Health Program; MND: mental and neurological disorders; PHC: primary health centre.

## 6 Conclusions

The enormous unmet need for care and support for hundreds of millions of children and adults with MNDs is now being increasingly acknowledged as a global health priority. One of the great challenges to scaling up services for people with MNDs has been the limited evidence of efficacious treatments and affordable delivery systems. The emerging evidence of the efficacy and cost-effectiveness of technically simple and affordable treatments delivered by non-specialist health workers is therefore a critically important evidence base for planning scaled-up programmes for people with MNDs.

The plan we have proposed is based on the socio-cultural, epidemiological and health system contexts of a specific location in one country. Although ‘one size does not fit all’ in health-system interventions, such a plan may serve as a blueprint for other contexts, following appropriate modification and adaptation to ensure its feasibility, acceptability and relevance. Thus, the recent WHO-WONCA review found that there was no single best practice model that can be followed by all countries for integration of mental health in primary care. Rather, “successes have been achieved through sensible local application of broad principles”.^26^ Although we believe that we have sufficient evidence to begin scaling up immediately, we accept the need for evidence to assess the impact of scaled-up interventions. Thus, there is a need for systematic documentation of the impact of such a plan on a range of indicators we have proposed. Success of this integrated programme would primarily be defined on its impact on closing the treatment gap for people with MNDs through improving access to evidence-based services that lead to improved clinical and functional outcomes. Ultimately, this evidence will be crucial, both to ensure that local programmes achieve optimal outcomes and to guide health policymakers who are still to embark on such programmes.

## Funding

VP is supported by a Wellcome Trust Senior Clinical Research Fellowship. The programme presented in this paper has been submitted to the Ministry of Health and Family Welfare, Government of India, for funding through its National Mental Health Program.

## Conflicts of interest

None declared.

## Ethical approval

Not required.

## Authors’ contributions

VP, DSG and RD all undertook all the duties of authorship; VP and RD designed the programme and DSG provided technical input. VP is guarantor of the paper.
